# Quantitative prediction of mouse class I MHC peptide binding affinity using support vector machine regression (SVR) models

**DOI:** 10.1186/1471-2105-7-182

**Published:** 2006-03-31

**Authors:** Wen Liu, Xiangshan Meng, Qiqi Xu, Darren R Flower, Tongbin Li

**Affiliations:** 1 Department of Neuroscience, University of Minnesota, Minneapolis, MN 55455, USA; 2 The Jenner Institute, University of Oxford, Compton, Berkshire RG20 7NN, UK

## Abstract

**Background:**

The binding between peptide epitopes and major histocompatibility complex proteins (MHCs) is an important event in the cellular immune response. Accurate prediction of the binding between short peptides and the MHC molecules has long been a principal challenge for immunoinformatics. Recently, the modeling of MHC-peptide binding has come to emphasize quantitative predictions: instead of categorizing peptides as "binders" or "non-binders" or as "strong binders" and "weak binders", recent methods seek to make predictions about precise binding affinities.

**Results:**

We developed a quantitative support vector machine regression (SVR) approach, called SVRMHC, to model peptide-MHC binding affinities. As a non-linear method, SVRMHC was able to generate models that out-performed existing linear models, such as the "additive method". By adopting a new "11-factor encoding" scheme, SVRMHC takes into account similarities in the physicochemical properties of the amino acids constituting the input peptides. When applied to MHC-peptide binding data for three mouse class I MHC alleles, the SVRMHC models produced more accurate predictions than those produced previously. Furthermore, comparisons based on Receiver Operating Characteristic (ROC) analysis indicated that SVRMHC was able to out-perform several prominent methods in identifying strongly binding peptides.

**Conclusion:**

As a method with demonstrated performance in the quantitative modeling of MHC-peptide binding and in identifying strong binders, SVRMHC is a promising immunoinformatics tool with not inconsiderable future potential.

## Background

The T cell, a specialized type of immune cell, continuously searches out proteins originating from pathogenic organisms, such as viruses, bacteria, fungi, or parasites. The T cell surface is enriched in a particular receptor protein: the T cell receptor or TCR, which binds to major histocompatibility complex proteins (MHCs) expressed on the surfaces of other cells. MHCs bind small peptide fragments derived from both host and pathogen proteins. It is the recognition of such complexes that lies at the heart of the cellular immune response. These short peptides are known as epitopes. Although the significance of non-peptide epitopes, such as lipids and carbohydrates, is now understood increasingly well, peptidic B cell and T cell epitopes (as mediated by the humoral and cellular immune systems respectively) remain the primary tools by which the intricate complexity of the immune response might be examined. While the prediction of B-cell epitopes remains primitive [1], a multiplicity of sophisticated methods for the prediction of T-cell epitopes has developed [2].

The earliest efforts in predicting the binding of short peptides to MHC molecules focused on identifying peptide sequence *motifs *that were characteristic of binding to MHC [3]. This *motif approach *assumed that the presence of certain residues at specific positions (which are referred to as "anchor" positions) critically defined the binding ability of the peptide to the MHC. This somewhat simplistic assumption rendered the *motif approach *prone to false predictions. Later methods adopted more informative representations of peptide binding and more sophisticated modeling strategies such as position-specific scoring matrices (PSSM) [4-7], artificial neural networks (ANN) [8-10], hidden Markov model (HMM) [11] and support vector machine (SVM) classification [12,13]. With increasing amounts of MHC-peptide binding data available to facilitate their optimization, these methods have become increasingly effective in making predictions about whether a given peptide binds to a particular MHC molecule, and – when it does bind – whether the binding is strong or weak.

Recently, the modeling of MHC-peptide binding has come to emphasize quantitative predictions: instead of categorizing peptides as "binders" or "non-binders" or as "strong binders" and "weak binders", several new methods make predictions about the precise binding affinities (usually expressed as pIC_50_, the negative logarithm of the IC_50_). The *additive method *developed by Doytchinova *et al*. is a representative example of this trend. In this method, the binding affinity of the MHC-peptide interaction is modeled as the sum of peptide background contribution (a constant term), the amino acid contributions at each position, and (optionally) the adjacent peptide side-chain interaction [14]. The additive method has been shown to be effective in modeling MHC-peptide binding for a range of human and mouse class I MHC molecules, and, using an iterative extension, also a set of human and mouse Class II alleles [14-17]. Additive method models not only provide more precise information about the binding reactions, but also demonstrated enhanced accuracy in the prediction of untested peptides compared to other prediction methods such as SYFPEITHI, BIMAS and RANKPEP [16,17].

In this paper, we shall explore how potential improvements might be made in quantitative immunoinformatic techniques, such as the additive method. First, utilizing non-linearity, since properly chosen non-linear models can, in describing complex systems, often out-perform linear models. Second, the use of a more informative scheme for encoding amino acids since most immunoinformatic methods encode amino acids by their identities using indicator variables, information concerning similarities in physicochemical properties between the 20 amino acids is typically neglected.

Support Vector Machines (SVMs) are a class of learning based non-linear modeling techniques with proven performance in a wide range of practical applications [18]. Originally, SVMs were developed for classification or qualitative modeling problems. With the introduction of an *ε*-insensitive loss function, SVMs have been extended to solve nonlinear regression (or quantitative modeling) problems. In this study, we employed the SVM regression (SVR) technique to model MHC-peptide binding affinities for three mouse class I MHC alleles (H2-Db, H2-Kb and H2-Kk). We name this new modeling method SVRMHC. In SVRMHC models, peptides were described using a new 11-factor encoding scheme. This takes into account a number of important physicochemical parameters of the 20 amino acids (including hydrophobicity scale, polarity, isoelectric point, and accessible surface area). These SVRMHC models demonstrated consistently better performance than linear methods in terms of describing power, self-consistency, and prediction accuracy. Moreover, comparisons between our SVR models and several other popular prediction tools indicated that the SVRMHC models performed best in identifying strong binders to mouse class I MHC molecules.

The datasets used in this study, and the online implementation of the SVRMHC models for the three mouse class I alleles, can be accessed on the supplementary web site [19].

## Results

### SVR model parameter optimization

For the training of the SVR models, one kernel parameter (*γ*), and two kernel-independent parameters (*ε *and *C*) need to be determined (Eq.(5)). There are no commonly agreed methods for determining optimal SVR model parameters. In most published SVR studies we have examined, these model parameters were determined one at a time, by first fixing all other parameters, then letting the parameter take a range of different values, and thus identifying the value that corresponds to the best model performance assessed by cross-validation [20,21]. This method, though efficient in terms of execution time, disregards potential interactions between different model parameters. Cherkassky and Ma [22] advocated picking two of the three SVR model parameters (*ε *and *C*) from training data based on characterizations of the data, such as noise level and sample number. This method, though theoretically sound, did not, in our hands, always find the best set of parameters. In this study, we adopted a parameter selection procedure that combines the method of Cherkassky and Ma's with a grid-search. For *ε *and *C*, we first calculated the "recommended value" using Cherkassky and Ma's formulas, then searched a parameter range from 1/10th of the recommended value to 10 times the recommended value. The kernel parameter*γ *does not depend on the datasets. We picked a search range for *γ *as [0.001, 1], which safely covered the *γ *ranges commonly used in the literature [20,21,23]. After setting search ranges for the three parameters, we undertook a grid-search through the three-dimensional parameter space. For each parameter, four, six or eight equal-sized steps (on logarithm scale) were taken in the grid-search.

**Figure 1:**
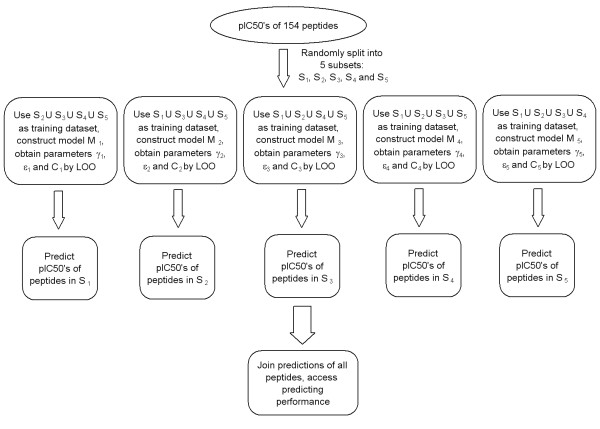
A schematic diagram of the five-fold cross-validation scheme for the training and testing of the SVRMHC model constructed for H2-Kk (154 peptides), with enclosing parameter searching modules in which leave-one-out (LOO) cross-validation was used. The models for the other two datasets (for H2-Db and H2-Kb) were constructed similarly, with the exception that the computationally more expensive LOO cross-validation (rather than five-fold cross-validation) was used on the outer-loop model training and testing procedure.

The H2-Kk dataset includes a large number of peptides (154 octamers), and five-fold cross-validation was performed to find the optimal parameters for the H2-Kk model (Figure 1). The other two datasets – H2-Db and H2-Kb – contain fewer peptides (65 nonamers and 62 octamers, respectively), thus a finer grained (and computationally more expensive) LOO cross-validation was used to search for optimal parameters for the models of these two MHC molecules. This LOO cross-validation is part of the model parameter search, and it should not be confused with the LOO cross-validation used in assessing the model performance (see *Methods*). The combination of the three parameters, *γ*, *ε *and *C *that leads to the smallest root mean square (RMS) error was taken as the optimal parameter combination. The RMS error is calculated as the following:

RMS=∑i=1n(pIC50i−pIC50i*)2n,     (1)
 MathType@MTEF@5@5@+=feaafiart1ev1aaatCvAUfKttLearuWrP9MDH5MBPbIqV92AaeXatLxBI9gBaebbnrfifHhDYfgasaacH8akY=wiFfYdH8Gipec8Eeeu0xXdbba9frFj0=OqFfea0dXdd9vqai=hGuQ8kuc9pgc9s8qqaq=dirpe0xb9q8qiLsFr0=vr0=vr0dc8meaabaqaciaacaGaaeqabaqabeGadaaakeaacqWGsbGucqWGnbqtcqWGtbWucqGH9aqpdaGcaaqaamaalaaabaWaaabCaeaacqGGOaakcqWGWbaCcqWGjbqscqWGdbWqcqaI1aqncqaIWaamdaWgaaWcbaGaemyAaKgabeaakiabgkHiTiabdchaWjabdMeajjabdoeadjabiwda1iabicdaWmaaDaaaleaacqWGPbqAaeaacqGGQaGkaaGccqGGPaqkdaahaaWcbeqaaiabikdaYaaaaeaacqWGPbqAcqGH9aqpcqaIXaqmaeaacqWGUbGBa0GaeyyeIuoaaOqaaiabd6gaUbaaaSqabaGccqGGSaalcaWLjaGaaCzcamaabmaabaGaeGymaedacaGLOaGaayzkaaaaaa@510E@

where *n *is the number of peptides in the dataset, *pIC*50_*i *_and pIC50i*
 MathType@MTEF@5@5@+=feaafiart1ev1aaatCvAUfKttLearuWrP9MDH5MBPbIqV92AaeXatLxBI9gBaebbnrfifHhDYfgasaacH8akY=wiFfYdH8Gipec8Eeeu0xXdbba9frFj0=OqFfea0dXdd9vqai=hGuQ8kuc9pgc9s8qqaq=dirpe0xb9q8qiLsFr0=vr0=vr0dc8meaabaqaciaacaGaaeqabaqabeGadaaakeaacqWGWbaCcqWGjbqscqWGdbWqcqaI1aqncqaIWaamdaqhaaWcbaGaemyAaKgabaGaeiOkaOcaaaaa@3489@ are the predicted and experimentally measured pIC50 values for the *i*th peptide, respectively. The final model parameters for the three MHC molecules were determined via voting among the set of optimal parameters during cross-validated model training. These parameters are presented in Table 1.

### SVRMHC models performed better than linear models in quantitative predictions

The SVRMHC models constructed for the three MHC molecules demonstrated consistently better performance than linear models built from the same datasets.

The H2-Db dataset consisted of 65 nonamer peptides and associated binding affinities. We compared the SVRMHC method to the additive model, taken as typical of linear methods, as shown in Table 2. Following a step-wise outlier exclusion procedure, the SVRMHC method determined and removed 3 outliers at a 2.0 log unit residual cut-off (see *Methods*), while the additive method removed 6. The smaller number of outliers determined by the SVRMHC method suggests that this method has more "descriptive power" than the additive method. The self-testing model constructed using the SVRMHC method resulted in an *r*^2 ^of 0.749 when the entire dataset was considered (including outliers), and an *r*^2 ^of 0.983 was obtained after the outliers were removed, compared to additive model *r*^2 ^values of 0.602 and 0.946, respectively. The AR values of the SVRMHC model with and without outliers were 0.170 and 0.043 respectively, smaller than the corresponding additive AR values of 0.403 and 0.187. The most interesting performance metric is perhaps the LOO cross-validated *q*^2^, as it is more indicative of prediction performance when tested on unseen data. *q*^2 ^for the SVRMHC method was 0.456. This is higher than the additive LOO cross-validated *q*^2 ^value of 0.401.

The H2-Kb dataset consisted of 62 octamer peptides and associated binding affinities (Table 2). With SVRMHC, the step-wise outlier exclusion procedure determined and excluded 6 outliers, compared to 7 outliers removed by the additive method. The self-testing model constructed using the SVRMHC method produced an *r*^2 ^of 0.568 for the entire dataset (including outliers), in contrast to the *r*^2 ^of 0.370 produced by the additive model. An *r*^2 ^of 0.970 was obtained with SVRMHC after the 6 outliers were excluded, which was lower than the *r*^2 ^obtained by the additive model (0.989) after the exclusion of 7 outliers. The AR of the SVRMHC model for the entire dataset (including outliers) was 0.382 and the AR of the additive method was 0.443. However, the AR of the SVM model after the 6 outliers were removed (0.130) was higher than the AR of the additive model after 7 outliers were removed (0.095). The LOO cross-validated *q*^2 ^of the model constructed with SVRMHC was 0.486, slightly higher than the additive LOO cross-validated *q*^2 ^of 0.454. These results indicated that for the H2-Kb dataset, SVRMHC produced models that had higher descriptive power and prediction accuracy, though the self-testing model exhibited lower level of self-consistency after the outliers were removed.

The H2-Kk dataset is the largest of the three datasets, consisting of 154 octamers and associated binding affinities. No outliers were excluded compared to 2 outliers using the additive method. This, again, suggests that the SVRMHC method has higher "descriptive power" than linear methods. The self-testing additive model produced an *r*^2 ^of 0.849 (whole dataset) and 0.933 (2 outliers excluded). The self-testing SVRMHC model gave an *r*^2 ^of 0.973. Since no outlier was determined, there is only one *r*^2 ^calculated. The AR for the SVRHMC model was 0.039, compared to the additive model, which gave 0.178 for the entire dataset and 0.151 after the outliers were removed. The LOO cross-validated *q*^2 ^for SVRMHC was 0.721, compared to an additive LOO cross-validated *q*^2 ^of 0.456.

### SVRMHC models out-performed other methods in identifying strong binders

We compared the performance of SVRMHC to that of existing prediction tools for MHC-peptide binding: the additive method, SYFPEITHI [5], BIMAS [6], RANKPEP [7], and SVMHC [12]. At first, we attempted a strategy described in [16,17]: trying to find recent literature reports of new peptide binding experiments for the three mouse class I MHC molecules. The hope was that predictions could be made for these binding experiments using both the SVRMHC model and the other methods, and a concomitant comparison in prediction accuracy could be made. However, this strategy was not successful, because in most recently-published binding experiments pre-screening with prediction tools was used. This was most often SYFPEITHI and sometimes BIMAS [24-29]. Only the peptides predicted to be strong binders were tested experimentally, and peptides not predicted to be strong binders were disregarded. Moreover, false predictions (peptides predicted by SYFPEITHI or BIMAS to be strong binders but which were determined experimentally to be weak binders or non-binders) were sometimes not reported [24,26,27]. It is not surprising that prediction tools used in pre-screening (SYFPEITHI or BIMAS) always performed better in identifying good binders in these published studies (results not shown).

Thus, we applied another scheme for making comparisons between the SVRMHC method and other prediction methods – by using Receiver Operating Characteristic (ROC) analysis [30,31]. The prediction performance of any classification-type model can be assessed using the combination of two properties – specificity and sensitivity. The *sensitivity *vs. (1-*specificity*) relationship is referred to as the ROC relationship (see *Methods*). In a ROC curve, the two axes both have a range of [0, 1], therefore the area under a ROC curve (*A*_*ROC*_) also takes a range of [0, 1]. A purely "random guess" prediction model would have an *A*_*ROC *_of 0.5. For better models, *A*_*ROC *_would be higher than 0.5. The closer *A*_*ROC *_is to 1, the better the performance is for the predicting model.

In the MHC ligand database MHCBN [32], we downloaded all nomamer ligands for the H2-Db molecule and all octamer ligands for H2-Kb and H2-Kk. These peptides were grouped into two groups: "strong binders" and "weak binders" (see *Methods*). For H2-Db, there were 28 strong binders and 44 weak binders, and for H2-Kb, there were 22 strong binders and 24 weak binders. No weak binders were retrieved for the H2-Kk, thus no ROC analysis was conducted for this allele. The scores generated by these different prediction methods have very different meanings. The scores produced by the SVRMHC models and the additive models are predicted pIC50 values, while scores from BIMAS are predicted half lives. RANKPEP outputs scores calculated from a PSSM (position-specific score matrix) profile. The scores produced by SYFPEITHI are nominal scores. They are generated by differentially scoring matches, within an individual peptide, to primary and secondary anchors within the target motif. Thus, they represent how close a particular peptide is to the expected pattern of a motif. The scores produced by SVMHC, on the other hand, are the distances between the peptides and the separating hyperplane defined by the SVM model. Despite the different meanings of these scores, they all roughly approximate increasing functions of predicted binding strength, therefore the areas under the ROC curves can be used as an objective measure of prediction performance.

**Figure 2:**
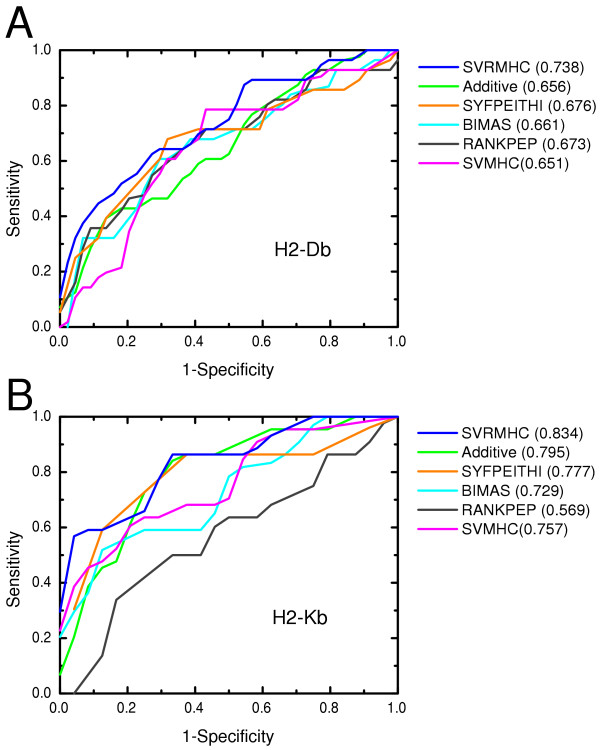
Comparison of the six predicting methods – SVRMHC, additive, SYFPEITHI, BIMAS, RANKPEP and SVMHC by ROC analysis (for H2-Db and H2-Dk). The ROC curves of different predicting methods are plotted in different colors. *A*_*ROC *_(area underneath the ROC curve) is provided following the label of each predicting method.

Predictions were made for each peptide sequence using the corresponding SVRMHC model, the corresponding additive model, as well as the four online predicting tools SYFPEITHI, BIMAS, RANKPEP and SVMHC, and the scores were used to make the ROC plots (Figure 2). The SVRMHC models for the H2-Kb and H2-Db molecules rendered *A*_*ROC *_of 0.738 and 0.834, respectively, higher than the *A*_*ROC *_of any of the other predicting methods, indicating that, in this test, the SVRMHC models performed best compared to the other four prediction methods in identifying strong binding peptides for the mouse class I MHC molecules.

## Discussion

Accurately predicting the binding between short peptides and MHC molecules remains a major task for immunoinformatics. Quantitative prediction of exact peptide binding affinity represents the most recent development in the field. Quantitative prediction is a finer-scale description of binding, and the ability to construct effective quantitative models manifests an improved understanding of the mechanism of MHC-peptide interactions. There have been two reported approaches to quantitative prediction of MHC-peptide binding. The first approach makes use of 3D QSAR (quantitative structure-activity relationship) techniques, and models the interaction between the peptide and the MHC molecule using CosMSIA (Comparative Molecular Similarity Indices Analysis) [33,34]. This approach, though accurate, requires structural knowledge about how the peptide and the MHC molecule interact with each other in 3D space. The second approach to quantitative modeling is the bioinformatics approach, including the additive method and the SVRMHC method presented here. In contrast to 3D-QSAR, this approach uses only peptide sequences as their input, and does not require any 3D structural information. This property makes the bioinformatics methods more straightforward and generally applicable. Particularly, they are more suitable for modeling the binding of less studied MHC molecules for which no 3D structural information is available.

Linear models, as exemplified here by the additive method, has previously demonstrated impressive performance in modeling a variety of MHC-peptide binding systems: the human class I allele HLA-A*0201 [14], the mouse Class I MHC alleles [17] and the human class II allele DRB1*0401 [15,16]. However, as is generally known, properly chosen non-linear models can often out-perform linear models in describing complex systems, although linear models can often be more intuitive and easily understood. Also, in many immunoinformatic techniques, including the additive method, amino acid residues are encoded by their identities, and the physicochemical properties of the amino acids are ignored. In this study, we have addressed both problems. The use of a non-linear SVR technique leads to an enhancement in predictivity. Meanwhile, the adoption of an 11-factor encoding of amino acids renders the resultant models sensitive to similarities in important physicochemical properties among the residues in the peptides being modeled.

Support vector machines (SVMs) are a new class of learning machines motivated by statistical learning theory [35], and they are gaining popularity because of their theoretically attractive features and profound empirical performance. Several reports have been seen in the literature where SVM classification models were developed to analyze peptide binding profiles qualitatively [12,13,36]; yet, to our knowledge, the current report is the first reported quantitative modeling study in which the SVR technique has been applied to model peptide binding.

In this study, we constructed SVRMHC models using the binding data of three mouse class I alleles (H2-Db, H2-Kb and H2-Kk), and compared the resulting models to a linear models, built using the additive method, constructed using the same datasets. The models constructed with SVRMHC have been shown to be superior to those constructed with linear methods, in terms of descriptive power (as shown by a smaller number of "outliers"), prediction accuracy (manifest as a higher cross-validated correlation coefficient *q*^2^), self-consistency (higher non-cross-validated explained variance *r*^2^), and overall precision in prediction (lower average residual of the prediction). Although an improved performance was seen in all three SVRMHC models, the levels of improvement differed between the models constructed for the three MHC alleles. There seems to be a positive correlation between the amount of improvement achieved by the SVRMHC models and dataset size. With the largest of the three datasets – the H2-Kk dataset (154 peptides), the SVRMHC method demonstrated the greatest level of improvement. The LOO cross-validated *q*^2 ^increased from 0.456 to 0.721. With the two smaller datasets – the H2-Db dataset (65 peptides) and the H2-Dk dataset (62 peptides) – the LOO cross-validated *q*^2 ^increased from 0.401 and 0.454 for the additive models to 0.456 and 0.486 for the SVRMHC models, respectively, marking a smaller improvement than for H2-Kk. When we looked at the self-consistency measure, with the two larger datasets (H2-Kk and H2-Db), the SVRMHC models consistently demonstrated higher levels of self-consistency than the linear models for the entire datasets as well as for the datasets after removal of outliers. For the smallest dataset, H2-Dk, although the SVRMHC model produced a higher *r*^2 ^than the corresponding linear model for the entire dataset (0.568 vs. 0.370); after outliers were removed, the SVRMHC model produced a lower *r*^2 ^than the additive model did (0.970 vs. 0.989). The same trend is true for the AR measurement. For the two larger datasets (H2-Kk and H2-Db), the SVRMHC models consistently produced lower AR values than the additive models for both the entire datasets and for the datasets after removal of outliers. However, for the smallest dataset (H2-Dk), the SVRMHC model produced a lower AR than the additive model for the entire dataset (0.382 vs. 0.443), but a higher AR than the additive model after the removal of outliers (0.130 vs. 0.095). These observations suggest that the SVRMHC approach may become more accurate as datasets grow.

In constructing the SVRMHC models, we applied the same step-wise outlier determination and exclusion scheme as used in [34] to ease the comparison between the SVRMHC and the additive methods. There are disagreements in the outliers determined by the two methods following the same step-wise outlier determining procedure (Table 4): 4 out of the 6 "outliers" determined by SVRMHC were also identified as "outliers" by the additive method for H2-Kb; only 1 out of the 3 "outliers" determined by SVRMHC was classified as an "outlier" by the additive method for H2-Db. These disagreements suggest that this outlier detection procedure may not be most accurate in identifying "true outliers" that reflect experimental errors. However, the main focus of this study is to demonstrate the performance of the SVRMHC method in comparison with other methods, therefore, it is justifiable to follow the same data pre-processing procedure as for the additive method for the sake of performance comparison. It is worth noting that after the model construction, the performance of the models was also examined on the whole dataset with the "outliers" added back, and SVRMHC consistently demonstrated higher accuracy than the linear method in the models constructed for all three alleles (see Average Residual (entire dataset), Table 2).

It is interesting to investigate whether the performance improvement of SVRMHC over the additive method is primarily due to the SVR modeling technique, or it is primarily attributed to the 11-factor encoding scheme for the peptide sequences. We constructed SVR models with the "sparse encoding" scheme following the same outlier exclusion procedure as used in the additive models and the SVRMHC models, and compared the three in their performance. As shown in Table 5, the "SVR + sparse encoding" method showed prediction performance that is between those of the additive and the SVRMHC methods for two of the three alleles – H2-Db and H2-Kk. For H2-Db, "SVR + sparse encoding" achieved a similar LOO cross-validated *q*^2 ^(0.459) to that of SVRMHC (0.456), but it excluded a larger number of outliers than SVRMHC (5 vs. 3). For H2-Kk, "SVR + sparse encoding" achieved a LOO cross-validated *q*^2 ^of 0.523, which is between the LOO cross-validated *q*^2 ^values of the additive (0.456) and SVRMHC method (0.721); and it excluded 1 outlier, also between the additive method (2 outliers excluded) and SVRMHC (no outlier excluded). This seems to suggest that both the SVR modeling technique and the 11-factor encoding scheme contributed to the superior performance of SVRMHC. However, we were surprised to see that the "SVR + sparse encoding" model for H2-Kb performed worse than both the SVRMHC model and the additive model (LOO cross-validated *q*^2 ^= 0.352, with 8 outliers removed), which is difficult to interpret. In order to be conclusive on this issue, models for a larger number of alleles need to be constructed and used in comparison; and this we intend to do in the near future.

The ROC analysis allows us to compare of several prediction tools for MHC-peptide binding: SVRMHC, the additive method, SYFPEITHI, BIMAS, RANKPEP and SVMHC. Our ROC analysis indicated that the SVRMHC method, with an average *A*_*ROC *_of 0.786, was the most accurate in identifying strong binders for the two mouse MHC molecules H2-Db and H2-Kb. It is followed by SYFPEITHI and the additive method (with average *A*_*ROC *_= 0.727 and 0.726, respectively). SVMHC (average *A*_*ROC *_= 0.704), BIMAS (average *A*_*ROC *_= 0.695) and RANKPEP (average *A*_*ROC *_= 0.621) were less accurate than the other three methods we compared. We need to stress, though, that this comparison is based on the models constructed for only two MHC molecules, and this rank order may not be true in the general case.

Questions may be raised about the fairness of the ROC-based comparison, because there are overlaps between the MHCBN data used in the ROC analysis and the data used in model construction for all five methods we compared. Ideally, a comparison based on a totally independent dataset, one with no overlaps with the data used in the model construction of any of the five methods, would be desirable. However, without information about what peptides were used in the model construction of the SYFPEITHI, BIMAS, RANKPEP and SVMHC methods, we believe it likely that there are higher levels of overlaps between the datasets used in model construction of the three qualitative models than those used for the two quantitative methods – additive and SVRMHC – because qualitative data are much more abundant than quantitative binding data. Nevertheless, we removed all peptides in the test datasets that overlapped with the datasets used in the construction of the additive and SVRMHC models (15 peptides for H2-Db, and 11 peptides for H2-Kb) and conducted a ROC-based comparison using the remaining data (Table 6). This is a very "unfair" comparison, because overlapped peptides for the additive and SVRMHC models were removed, but those for the other methods were not. Yet, the results indicated that the SVRMHC model for H2-Kb (*A*_*ROC *_= 0.83) still out-performed the models for all other methods; and the SVRMHC model for H2-Db, though did not achieve as high *A*_*ROC *_as BIMAS (0.66) or RANKPEP (0.677), but was still close to them (0.658).

Despite their encouraging performance, SVR-based models reported here also exhibit some disadvantages. Most notably, these models are "black box" models, and are poorly interpretable. We cannot infer, for example, which peptide positions are the most important in determining the strength of the MHC-peptide binding. Not that this necessarily obviates the utility of SVRMHC models as an immunological tool.

Currently, we are working to improve further SVR-based modeling methods, focusing on testing different combinations of physicochemical properties in the feature encoding scheme. We also plan to construct MHC-peptide binding models for other MHC molecules hosted in the AntiJen database [37,38] and to make these prediction models available online. In the next phase of this project, we will adapt the SVR-based methodology to the more challenging task of predicting the MHC-peptide binding of class II MHCs.

## Conclusion

In this paper, we demonstrated SVRMHC, a SVR-based quantitative modeling approach to model peptide-MHC binding affinities, and showed that SVRMHC is a promising immunoinformatics tool with not inconsiderable future potential. With the ongoing, rapid development of high-throughput functional proteomics technologies, such as peptide microarray technology, the SVR modeling approach is expected to see broader use in modeling MHC-peptide binding, and protein-peptide binding reactions in general.

## Methods

### Support Vector Machine Regression (SVR) overview

Support Vector Machines (SVMs) are a class of learning machines based on statistical learning theory [18,35]. With the introduction of an *ε*-insensitive loss function, SVMs have been extended to solve nonlinear regression estimation [35]. In SVR, with input data set G={(xi,di)}in
 MathType@MTEF@5@5@+=feaafiart1ev1aaatCvAUfKttLearuWrP9MDH5MBPbIqV92AaeXatLxBI9gBaebbnrfifHhDYfgasaacH8akY=wiFfYdH8Gipec8Eeeu0xXdbba9frFj0=OqFfea0dXdd9vqai=hGuQ8kuc9pgc9s8qqaq=dirpe0xb9q8qiLsFr0=vr0=vr0dc8meaabaqaciaacaGaaeqabaqabeGadaaakeaacqWGhbWrcqGH9aqpcqGG7bWEcqGGOaakcqWG4baEdaWgaaWcbaGaemyAaKgabeaakiabcYcaSiabdsgaKnaaBaaaleaacqWGPbqAaeqaaOGaeiykaKIaeiyFa03aa0baaSqaaiabdMgaPbqaaiabd6gaUbaaaaa@3D34@ (where *x*_*i *_is the input vector, *d*_*i *_is the desired real-valued labeling, and *n *is the number of the input records), *x *is first mapped into a higher-dimension feature space *F *via a nonlinear mappingΘ, then linear regression is performed in this space. In other words, SVR approximate a function using the following equation

*y *= *f*(*x*) = *w*Θ(*x*) + *b *    (2)

The coefficients *w*and *b *are estimated by minimizing

R(C)=12‖w‖2+C1n∑i=1nLε(di,yi)     (3)
 MathType@MTEF@5@5@+=feaafiart1ev1aaatCvAUfKttLearuWrP9MDH5MBPbIqV92AaeXatLxBI9gBaebbnrfifHhDYfgasaacH8akY=wiFfYdH8Gipec8Eeeu0xXdbba9frFj0=OqFfea0dXdd9vqai=hGuQ8kuc9pgc9s8qqaq=dirpe0xb9q8qiLsFr0=vr0=vr0dc8meaabaqaciaacaGaaeqabaqabeGadaaakeaacqWGsbGucqGGOaakcqWGdbWqcqGGPaqkcqGH9aqpdaWcaaqaaiabigdaXaqaaiabikdaYaaadaqbdaqaaiabdEha3bGaayzcSlaawQa7amaaCaaaleqabaGaeGOmaidaaOGaey4kaSIaem4qam0aaSaaaeaacqaIXaqmaeaacqWGUbGBaaWaaabCaeaacqWGmbatdaWgaaWcbaacciGae8xTdugabeaaaeaacqWGPbqAcqGH9aqpcqaIXaqmaeaacqWGUbGBa0GaeyyeIuoakiabcIcaOiabdsgaKnaaBaaaleaacqWGPbqAaeqaaOGaeiilaWIaemyEaK3aaSbaaSqaaiabdMgaPbqabaGccqGGPaqkcaWLjaGaaCzcamaabmaabaGaeG4mamdacaGLOaGaayzkaaaaaa@53E2@

where *L*_*ε *_(*d*, *y*) is the empirical error measured by *ε*-insensitive loss function

Lε(d,y)={|d−y|−ε,if|d−y|≥00,otherwise,     (4)
 MathType@MTEF@5@5@+=feaafiart1ev1aaatCvAUfKttLearuWrP9MDH5MBPbIqV92AaeXatLxBI9gBaebbnrfifHhDYfgasaacH8akY=wiFfYdH8Gipec8Eeeu0xXdbba9frFj0=OqFfea0dXdd9vqai=hGuQ8kuc9pgc9s8qqaq=dirpe0xb9q8qiLsFr0=vr0=vr0dc8meaabaqaciaacaGaaeqabaqabeGadaaakeaacqWGmbatdaWgaaWcbaacciGae8xTdugabeaakiabcIcaOiabdsgaKjabcYcaSiabdMha5jabcMcaPiabg2da9maaceaabaqbaeqabiqaaaqaaiabcYha8jabdsgaKjabgkHiTiabdMha5jabcYha8jabgkHiTiab=v7aLjabcYcaSiabdMgaPjabdAgaMjabcYha8jabdsgaKjabgkHiTiabdMha5jabcYha8jabgwMiZkabicdaWaqaaiabicdaWiabcYcaSiabd+gaVjabdsha0jabdIgaOjabdwgaLjabdkhaYjabdEha3jabdMgaPjabdohaZjabdwgaLbaaaiaawUhaaiabcYcaSiaaxMaacaWLjaWaaeWaaeaacqaI0aanaiaawIcacaGLPaaaaaa@6077@

and the term 1/2||*w*||^2 ^is a regularization term. The constant *C *is specified by the user, and it determines the trade-off between the empirical risk and the regularization term. *ε *is also specified by the user, and it is equivalent to the approximation accuracy of the training data.

The estimations of *w *and *b *are obtained by transforming Eq. (3) into the primal function:

R(w,ξ(*))=12‖w‖2+C∑i=1n(ξi+ξi*)
 MathType@MTEF@5@5@+=feaafiart1ev1aaatCvAUfKttLearuWrP9MDH5MBPbIqV92AaeXatLxBI9gBaebbnrfifHhDYfgasaacH8akY=wiFfYdH8Gipec8Eeeu0xXdbba9frFj0=OqFfea0dXdd9vqai=hGuQ8kuc9pgc9s8qqaq=dirpe0xb9q8qiLsFr0=vr0=vr0dc8meaabaqaciaacaGaaeqabaqabeGadaaakeaacqWGsbGucqGGOaakcqWG3bWDcqGGSaaliiGacqWF+oaEdaahaaWcbeqaaiabcIcaOiabcQcaQiabcMcaPaaakiabcMcaPiabg2da9maalaaabaGaeGymaedabaGaeGOmaidaamaafmaabaGaem4DaChacaGLjWUaayPcSdWaaWbaaSqabeaacqaIYaGmaaGccqGHRaWkcqWGdbWqdaaeWbqaaiabcIcaOiab=57a4naaBaaaleaacqWGPbqAaeqaaOGaey4kaSIae8NVdG3aa0baaSqaaiabdMgaPbqaaiabcQcaQaaakiabcMcaPaWcbaGaemyAaKMaeyypa0JaeGymaedabaGaemOBa4ganiabggHiLdaaaa@5228@

subject to{di−wΘ(xi)−bi≤ε+ξiwΘ(xi)+bi−di≤ε+ξi*ξi,ξi*≥0     (5)
 MathType@MTEF@5@5@+=feaafiart1ev1aaatCvAUfKttLearuWrP9MDH5MBPbIqV92AaeXatLxBI9gBaebbnrfifHhDYfgasaacH8akY=wiFfYdH8Gipec8Eeeu0xXdbba9frFj0=OqFfea0dXdd9vqai=hGuQ8kuc9pgc9s8qqaq=dirpe0xb9q8qiLsFr0=vr0=vr0dc8meaabaqaciaacaGaaeqabaqabeGadaaakeaacqqGZbWCcqqG1bqDcqqGIbGycqqGQbGAcqqGLbqzcqqGJbWycqqG0baDcqqGGaaicqqG0baDcqqGVbWBdaGabaqaauaabeqadeaaaeaacqWGKbazdaWgaaWcbaGaemyAaKgabeaakiabgkHiTiabdEha3jabfI5arjabcIcaOiabdIha4naaBaaaleaacqWGPbqAaeqaaOGaeiykaKIaeyOeI0IaemOyai2aaSbaaSqaaiabdMgaPbqabaGccqGHKjYOiiGacqWF1oqzcqGHRaWkcqWF+oaEdaWgaaWcbaGaemyAaKgabeaaaOqaaiabdEha3jabfI5arjabcIcaOiabdIha4naaBaaaleaacqWGPbqAaeqaaOGaeiykaKIaey4kaSIaemOyai2aaSbaaSqaaiabdMgaPbqabaGccqGHsislcqWGKbazdaWgaaWcbaGaemyAaKgabeaakiabgsMiJkab=v7aLjabgUcaRiab=57a4naaDaaaleaacqWGPbqAaeaacqGGQaGkaaaakeaacqWF+oaEdaWgaaWcbaGaemyAaKgabeaakiabcYcaSiab=57a4naaDaaaleaacqWGPbqAaeaacqGGQaGkaaGccqGHLjYScqaIWaamaaaacaGL7baacaWLjaGaaCzcamaabmaabaGaeGynaudacaGLOaGaayzkaaaaaa@784B@

By introducing Lagrange multipliers, the optimization problem can be transformed into a quadratic programming problem. The solution takes the following form:

f(x,ai,ai*)=∑(ai−ai*)K(x,xi)+b     (6)
 MathType@MTEF@5@5@+=feaafiart1ev1aaatCvAUfKttLearuWrP9MDH5MBPbIqV92AaeXatLxBI9gBaebbnrfifHhDYfgasaacH8akY=wiFfYdH8Gipec8Eeeu0xXdbba9frFj0=OqFfea0dXdd9vqai=hGuQ8kuc9pgc9s8qqaq=dirpe0xb9q8qiLsFr0=vr0=vr0dc8meaabaqaciaacaGaaeqabaqabeGadaaakeaacqWGMbGzcqGGOaakcqWG4baEcqGGSaalcqWGHbqydaWgaaWcbaGaemyAaKgabeaakiabcYcaSiabdggaHnaaDaaaleaacqWGPbqAaeaacqGGQaGkaaGccqGGPaqkcqGH9aqpdaaeabqaaiabcIcaOiabdggaHnaaBaaaleaacqWGPbqAaeqaaOGaeyOeI0Iaemyyae2aa0baaSqaaiabdMgaPbqaaiabcQcaQaaakiabcMcaPiabdUealjabcIcaOiabdIha4jabcYcaSiabdIha4naaBaaaleaacqWGPbqAaeqaaOGaeiykaKIaey4kaSIaemOyaiMaaCzcaiaaxMaadaqadaqaaiabiAda2aGaayjkaiaawMcaaaWcbeqab0GaeyyeIuoaaaa@53F9@

where *K *is the kernel function *K*(*x*, *x*_*i*_) = Θ(*x*)^*T *^Θ(*x*_*i*_) By using of a kernel function, we can deal with problems of arbitrary dimensionality without having to compute the mapping Θ explicitly. Commonly used kernels include the linear kernel, polynomial kernel, and the radial basis function (RBF) kernel. In this exploration, we chose to use the RBF (radial basis function) kernel as recommended in Chang and Lin [39]. The RBF kernel takes the following form:

*K*(*x*_*i*_, *x*_*i*_) = exp(-*γ*||*x *- *x*_*i*_||^2^), *γ *> 0.     (7)

### Data description

We constructed SVRMHC models using three MHC-peptide binding datasets for mouse Class I MHC alleles. These sets have been used previously to construct models using the additive method [17], facilitating comparison between the two methods. The data consists of peptide sequences and experimentally measured binding affinities (expressed numerically as pIC50). The first dataset contains 65 nonamer peptides (H2-Db allele), the second dataset 62 octamers (H2-Kb), and the third dataset 154 octamer peptides (H2-Kk).

### Encoding scheme of peptide sequences

The most widely-used representation of an amino acid sequence in immunoinformatic modelling is the "sparse encoding" scheme [12,40]. However, such an encoding scheme does not account for any similarity in physicochemical properties between amino acids. We developed a new encoding method. First, from AA-index [41], we picked a list of what we considered as important general physicochemical properties (e.g., polarity, isoelectric point, and accessible surface area). Into this list, we added a number of properties that were identified in 3-D QSAR analysis [34] as key determinants of peptide-MHC interaction (volume, number of hydrogen bond donors, hydrophobicity). This led to a list of properties that consists of 17 physicochemical indices. We calculated the pair-wise correlation coefficients (*r*^2^) of these 17 factors. For any pair of factors with *r*^2 ^> 0.8, we eliminated one of the two factors. In the end, a list of 11 factors was obtained. The values of the 11 physicochemical parameters were linearly scaled to the range [0, 1] for the 22 amino acids (Table 5). The list of the 17 factors and their pair-wise *r*^2 ^are presented in online supplementary material [19]. As input to the SVRMHC models, a given octamer or nonamer peptide sequence is represented as a long vector concatenated from the eight or nine numerical vectors (each of length 11) encoding the corresponding residue in the sequence. We name this encoding scheme the "11-factor encoding".

### Outlier determination and exclusion

To ease comparison of SVRMHC models and those constructed previously using the additive method, we applied the same step-wise outlier determination and exclusion scheme as used in [34]. For each dataset, a SVRMHC model was first constructed using the whole dataset, and prediction was made for each sequence in the whole dataset. We called this model the "self-testing model". If at least one sequence in the dataset produced a residual value = 2.0 log units in the "self-testing model" (the residual value is defined as the absolute value of the difference between the predicted affinity and true affinity on logarithm scale), then the sequence with the maximum residual value was excluded as an outlier, and a replacement self-testing model was constructed using the remaining sequences. This procedure was repeated until all sequences in the dataset had residual values < 2.0 log units.

### Assessment of model performance

The performance of the SVRMHC models was assessed using several metrics. The number of outliers determined and excluded can be considered as a measurement of "descriptive power" of a model: a model that excludes a smaller number of outliers is better at describing the dataset as a whole than a model that excludes a greater number of outliers. For the final self-testing model (the self-testing model after all outliers are removed), we can assess its "self-consistency" using the explained variance (or squared correlation coefficient) *r*^2 ^(see [17]).

The most important measure of a model's performance is its prediction accuracy, which can be assessed by the cross-validated correlation coefficient, *q*^2^, of the model:

q2=1−∑i=1n(pIC50i−pIC50i*)2∑i=1n(pIC50i*−pIC50*¯)2,     (8)
 MathType@MTEF@5@5@+=feaafiart1ev1aaatCvAUfKttLearuWrP9MDH5MBPbIqV92AaeXatLxBI9gBaebbnrfifHhDYfgasaacH8akY=wiFfYdH8Gipec8Eeeu0xXdbba9frFj0=OqFfea0dXdd9vqai=hGuQ8kuc9pgc9s8qqaq=dirpe0xb9q8qiLsFr0=vr0=vr0dc8meaabaqaciaacaGaaeqabaqabeGadaaakeaacqWGXbqCdaahaaWcbeqaaiabikdaYaaakiabg2da9iabigdaXiabgkHiTmaalaaabaWaaabCaeaacqGGOaakcqWGWbaCcqWGjbqscqWGdbWqcqaI1aqncqaIWaamdaWgaaWcbaGaemyAaKgabeaakiabgkHiTiabdchaWjabdMeajjabdoeadjabiwda1iabicdaWmaaDaaaleaacqWGPbqAaeaacqGGQaGkaaGccqGGPaqkdaahaaWcbeqaaiabikdaYaaaaeaacqWGPbqAcqGH9aqpcqaIXaqmaeaacqWGUbGBa0GaeyyeIuoaaOqaamaaqahabaGaeiikaGIaemiCaaNaemysaKKaem4qamKaeGynauJaeGimaaZaa0baaSqaaiabdMgaPbqaaiabcQcaQaaakiabgkHiTmaanaaabaGaemiCaaNaemysaKKaem4qamKaeGynauJaeGimaaZaaWbaaSqabeaacqGGQaGkaaaaaOGaeiykaKYaaWbaaSqabeaacqaIYaGmaaaabaGaemyAaKMaeyypa0JaeGymaedabaGaemOBa4ganiabggHiLdaaaOGaeiilaWIaaCzcaiaaxMaadaqadaqaaiabiEda3aGaayjkaiaawMcaaaaa@69BB@

where *n *is the number of peptides in the dataset, *pIC*50_*i *_and pIC50i*
 MathType@MTEF@5@5@+=feaafiart1ev1aaatCvAUfKttLearuWrP9MDH5MBPbIqV92AaeXatLxBI9gBaebbnrfifHhDYfgasaacH8akY=wiFfYdH8Gipec8Eeeu0xXdbba9frFj0=OqFfea0dXdd9vqai=hGuQ8kuc9pgc9s8qqaq=dirpe0xb9q8qiLsFr0=vr0=vr0dc8meaabaqaciaacaGaaeqabaqabeGadaaakeaacqWGWbaCcqWGjbqscqWGdbWqcqaI1aqncqaIWaamdaqhaaWcbaGaemyAaKgabaGaeiOkaOcaaaaa@3489@ are the predicted and experimentally measured pIC50 values for the *i*th peptide, respectively, and pIC50*¯
 MathType@MTEF@5@5@+=feaafiart1ev1aaatCvAUfKttLearuWrP9MDH5MBPbIqV92AaeXatLxBI9gBaebbnrfifHhDYfgasaacH8akY=wiFfYdH8Gipec8Eeeu0xXdbba9frFj0=OqFfea0dXdd9vqai=hGuQ8kuc9pgc9s8qqaq=dirpe0xb9q8qiLsFr0=vr0=vr0dc8meaabaqaciaacaGaaeqabaqabeGadaaakeaadaqdaaqaaiabdchaWjabdMeajjabdoeadjabiwda1iabicdaWmaaCaaaleqabaGaeiOkaOcaaaaaaaa@333F@ is the mean of the experimentally measured pIC50 values. As in [17], we used leave-one-out (LOO) cross-validation to check our models' prediction performance.

Another metric that can be used to assess the performance of the models is the average residual (AR), defined simply as

AR=∑i=1n|pIC50i−pIC50i*|.     (9)
 MathType@MTEF@5@5@+=feaafiart1ev1aaatCvAUfKttLearuWrP9MDH5MBPbIqV92AaeXatLxBI9gBaebbnrfifHhDYfgasaacH8akY=wiFfYdH8Gipec8Eeeu0xXdbba9frFj0=OqFfea0dXdd9vqai=hGuQ8kuc9pgc9s8qqaq=dirpe0xb9q8qiLsFr0=vr0=vr0dc8meaabaqaciaacaGaaeqabaqabeGadaaakeaacqWGbbqqcqWGsbGucqGH9aqpdaaeWbqaamaaemaabaGaemiCaaNaemysaKKaem4qamKaeGynauJaeGimaaZaaSbaaSqaaiabdMgaPbqabaGccqGHsislcqWGWbaCcqWGjbqscqWGdbWqcqaI1aqncqaIWaamdaqhaaWcbaGaemyAaKgabaGaeiOkaOcaaaGccaGLhWUaayjcSdaaleaacqWGPbqAcqGH9aqpcqaIXaqmaeaacqWGUbGBa0GaeyyeIuoakiabc6caUiaaxMaacaWLjaWaaeWaaeaacqaI4aaoaiaawIcacaGLPaaaaaa@4E9B@

The AR is a measure of the overall precision of the prediction made by the model. A model with a lower AR overall makes more precise prediction than a model with a higher AR.

### ROC analysis and comparisons of SVR models with other predicting tools

Prediction performance of any classification-type model can be assessed by the combination of two parameters: "false positive rate" and the "false negative rate" or, equivalently, *specificity *and *sensitivity*. *Sensitivity *is defined as 1- "false negative rate", and *specificity *is defined as the 1- "false positive rate". A plot of *sensitivity *vs. (1-*specificity*) is known as the ROC curve.

In the MHC ligand database MHCBN [32], all nomamer ligands for the H2-Db molecule and all octamer ligands for H2-Kb and H2-Kk were downloaded. In the MHCBN database, the peptide ligands are classified into five categories: "high binding", "moderate binding", "low binding", "no-binding" and "unknown". We grouped all peptides in the "high binding" and "moderate binding" categories together as "strong binders", all peptides in the "low binding" and "no-binding" categories together as "weak binders", and discarded the peptides in the "unknown" category. All ligands for the H2-Kk molecule downloaded from MHCBN were "strong binders", therefore the ROC analysis was not performed with H2-Kk.

The scores used for the SVRMHC method in the ROC analysis were the predicted pIC_50 _values of the test ligands for the final SVRMHC models. The scores used for the additive method [42], SYFPEITHI [43], BIMAS [44], RANKPEP [45], and SVMHC [46] were obtained by querying the corresponding online predicting servers. Default parameters were used when making the queries. After the scores of all peptides for a MHC molecule (H2-Db or H2-Kb) were obtained, each score value was used in turn as a cut-off point. At each cut-off point s⌢
 MathType@MTEF@5@5@+=feaafiart1ev1aaatCvAUfKttLearuWrP9MDH5MBPbIqV92AaeXatLxBI9gBaebbnrfifHhDYfgasaacH8akY=wiFfYdH8Gipec8Eeeu0xXdbba9frFj0=OqFfea0dXdd9vqai=hGuQ8kuc9pgc9s8qqaq=dirpe0xb9q8qiLsFr0=vr0=vr0dc8meaabaqaciaacaGaaeqabaqabeGadaaakeaacuWGZbWCgaWeaaaa@2E35@, the true positive rate was calculated as

rt,p=#{i|si≥s⌢,i∈S)#{i|si≥s⌢),     (10)
 MathType@MTEF@5@5@+=feaafiart1ev1aaatCvAUfKttLearuWrP9MDH5MBPbIqV92AaeXatLxBI9gBaebbnrfifHhDYfgasaacH8akY=wiFfYdH8Gipec8Eeeu0xXdbba9frFj0=OqFfea0dXdd9vqai=hGuQ8kuc9pgc9s8qqaq=dirpe0xb9q8qiLsFr0=vr0=vr0dc8meaabaqaciaacaGaaeqabaqabeGadaaakeaacqWGYbGCdaWgaaWcbaGaemiDaqNaeiilaWIaemiCaahabeaakiabg2da9maalaaabaGaei4iamIaei4EaSNaemyAaKMaeiiFaWNaem4Cam3aaSbaaSqaaiabdMgaPbqabaGccqGHLjYScuWGZbWCgaWeaiabcYcaSiabdMgaPjabgIGiolabdofatjabcMcaPaqaaiabcocaJiabcUha7jabdMgaPjabcYha8jabdohaZnaaBaaaleaacqWGPbqAaeqaaOGaeyyzImRafm4CamNbambacqGGPaqkaaGaeiilaWIaaCzcaiaaxMaadaqadaqaaiabiMda5aGaayjkaiaawMcaaaaa@555A@

where *s_i _*is the predicted score for peptide *i*, and *S *is the set of all "strong binders". The false positive rate was calculated as

rf,p=#{i|si≥s⌢,i∈W)#{i|si≥s⌢),     (11)
 MathType@MTEF@5@5@+=feaafiart1ev1aaatCvAUfKttLearuWrP9MDH5MBPbIqV92AaeXatLxBI9gBaebbnrfifHhDYfgasaacH8akY=wiFfYdH8Gipec8Eeeu0xXdbba9frFj0=OqFfea0dXdd9vqai=hGuQ8kuc9pgc9s8qqaq=dirpe0xb9q8qiLsFr0=vr0=vr0dc8meaabaqaciaacaGaaeqabaqabeGadaaakeaacqWGYbGCdaWgaaWcbaGaemOzayMaeiilaWIaemiCaahabeaakiabg2da9maalaaabaGaei4iamIaei4EaSNaemyAaKMaeiiFaWNaem4Cam3aaSbaaSqaaiabdMgaPbqabaGccqGHLjYScuWGZbWCgaWeaiabcYcaSiabdMgaPjabgIGiolabdEfaxjabcMcaPaqaaiabcocaJiabcUha7jabdMgaPjabcYha8jabdohaZnaaBaaaleaacqWGPbqAaeqaaOGaeyyzImRafm4CamNbambacqGGPaqkaaGaeiilaWIaaCzcaiaaxMaadaqadaqaaiabigdaXiabicdaWaGaayjkaiaawMcaaaaa@5624@

where *W *is the set of all "weak binders". The ROC curve was plotted as *r*_*f*,*p *_vs. *r*_*t*,*p*_.

## Authors' contributions

WL carried out the detailed design for the major components of this study, and participated in the coding and computing work. XM executed the major parts of the coding and computing. QX participated in the performance assessment work and comparisons between SVRMHC and other methods, and constructed the supplementary web site with online SVRMHC implementation. DRF provided the data for constructing the SVRMHC models, as well as significant assistance and advice on essential issues of the model construction, and participated in the writing of the manuscript. TL conceived of and coordinated the study, participated in the design, and drafted the manuscript.

## Figures and Tables

**Table 1:** The parameters recommended by Cherkassky and Ma (2004) (*ε *and C) and the final optimized parameters (*ε*, C and *γ*) of the SVRMHC models constructed for the three mouse class I alleles.

	***ε*-recommended**	***ε*-optimized**	**C-recommended**	**C-optimized**	***γ*-optimized**
**Model for H2-Db**	0.0475	0.0150	10.34	18.39	0.0316
**Model for H2-Kb**	0.0513	0.5134	10.88	34.41	0.0316
**Model for H2-Kk**	0.0152	0.0152	10.00	10.00	0.3162

**Table 2:** Comparison between the additive method and the SVRMHC method in models constructed with the H2-Db, H2-Kb and H2-Kk datasets.

	**H2-Db**		**H2-Kb**		**H2-Kk**	
	
	Additive Method	SVRMHC Method	Additive Method	SVRMHC Method	Additive Method	SVRMHC Method
**Numbers of outliers**	6	3	7	6	2	0
***r*^2 ^(self-consistency, outliers removed)**	0.946	0.983	0.989	0.97	0.933	0.973
***r*^2 ^(self-consistency, entire dataset)**	0.602	0.749	0.37	0.568	0.849	0.973
**Average Residual (entire dataset)**	0.403	0.17	0.443	0.382	0.178	0.039
**Average Residual (outliers removed)**	0.187	0.043	0.095	0.13	0.151	0.039
***q*^2 ^(LOO_CV) (outliers removed)**	0.401	0.456	0.454	0.486	0.456	0.721

**Table 3:** The scores used in the 11-factor encoding for the 20 amino acids, after scaling to the range [0, 1].

**Amino Acid**	**Steric parameter**	**Hydrogen bond donors**	**Hydropho-bicity scale**	**Hydrophi-licity scale**	**Average accessible surface area**	**van der Waals parameter R0**	**van der Waals parameter epsilon**	**Free energy of solution in water**	**Average side chain orientation angle**	**Polarity**	**Isoelectric point**
**A**	0.510	0.169	0.471	0.279	0.141	0.294	0.000	0.262	0.512	0.000	0.404
**R**	0.667	0.726	0.321	1.000	0.905	0.529	0.327	0.169	0.372	1.000	1.000
**N**	0.745	0.390	0.164	0.658	0.510	0.235	0.140	0.313	0.116	0.065	0.330
**D**	0.745	0.304	0.021	0.793	0.515	0.235	0.140	0.601	0.140	0.956	0.000
**C**	0.608	0.314	0.760	0.072	0.000	0.559	0.140	0.947	0.907	0.028	0.285
**Q**	0.667	0.531	0.178	0.649	0.608	0.529	0.140	0.416	0.023	0.068	0.360
**E**	0.667	0.482	0.092	0.883	0.602	0.529	0.140	0.561	0.163	0.960	0.056
**G**	0.000	0.000	0.275	0.189	0.103	0.000	0.000	0.240	0.581	0.000	0.401
**H**	0.686	0.554	0.326	0.468	0.402	0.529	0.140	0.313	0.581	0.992	0.603
**I**	1.000	0.650	1.000	0.000	0.083	0.824	0.308	0.424	0.930	0.003	0.407
**L**	0.961	0.650	0.734	0.081	0.138	0.824	0.308	0.463	0.907	0.003	0.402
**K**	0.667	0.692	0.000	0.568	1.000	0.529	0.327	0.313	0.000	0.952	0.872
**M**	0.765	0.612	0.603	0.171	0.206	0.765	0.308	0.405	0.814	0.028	0.372
**F**	0.686	0.772	0.665	0.000	0.114	0.853	0.682	0.462	1.000	0.007	0.339
**P**	0.353	0.372	0.012	0.198	0.411	0.588	0.271	0.000	0.302	0.030	0.442
**S**	0.520	0.172	0.155	0.477	0.303	0.206	0.000	0.240	0.419	0.032	0.364
**T**	0.490	0.349	0.256	0.523	0.337	0.235	0.140	0.313	0.419	0.032	0.362
**W**	0.686	1.000	0.681	0.207	0.219	1.000	1.000	0.537	0.674	0.040	0.390
**Y**	0.686	0.796	0.591	0.477	0.454	0.853	0.682	1.000	0.419	0.031	0.362
**V**	0.745	0.487	0.859	0.036	0.094	0.647	0.234	0.369	0.674	0.003	0.399

**Table 4:** The outliers determined by the additive method and the SVRMHC method for H2-Db, H2-Kb and H2-Kk. Common outliers determined by both methods are italicized.

**H2-Db**							
**Outliers determined by Additive method**				**Outliers determined by SVRMHC method**			

	True *pIC50*	Predicted *pIC50 *(Additive)	Predicted *pIC50 *(SVRMHC)		True *pIC50*	Predicted *pIC50 *(Additive)	Predicted *pIC50 *(SVRMHC)
*QLPPNSLLI*	*3.53*	*6.19*	*7.06*	*QLPPNSLLI*	*3.53*	*6.19*	*7.06*
GFKSNFNKI	3.36	6.30	5.28	TAGANPMDL	4.66	4.84	7.30
IKPSNSEDL	5.54	7.70	6.33	CKGVNKEYL	7.41	7.13	5.14
TALANTIEV	8.44	5.75	7.02				
TGKLNLENL	4.75	7.10	6.47				
AEDTNVSLI	3.36	5.73	4.62				
**H2-Kb**							

**Outliers determined by Additive method**				**Outliers determined by SVRMHC method**			

	True *pIC50*	Predicted *pIC50 *(Additive)	Predicted *pIC50 *(SVRMHC)		True *pIC50*	Predicted *pIC50 *(Additive)	Predicted *pIC50 *(SVRMHC)
*NTVVFDAL*	*3.81*	*6.97*	*7.44*	*NTVVFDAL*	*3.81*	*6.97*	*7.44*
*LQQRYSRL*	*9.22*	*5.80*	*6.42*	*LQQRYSRL*	*9.22*	*5.80*	*6.42*
*SKLQYKII*	*3.81*	*6.96*	*6.66*	*SKLQYKII*	*3.81*	*6.96*	*6.66*
*QPQNYLRL*	*4.29*	*9.49*	*6.61*	*QPQNYLRL*	*4.29*	*9.49*	*6.61*
MGLIYNRM	8.34	6.21	7.56	VLLDYQGM	5.48	5.62	7.95
IIFLFILL	5.13	7.85	6.36	SIILFLPL	9.00	8.81	6.72
MWYWGPSL	5.13	7.58	7.11				
**H2-Kk**							

**Outliers determined by Additive method**				**Outliers determined by SVRMHC method**			

	True *pIC50*	Predicted *pIC50 *(Additive)	Predicted *pIC50 *(SVRMHC)	(none)			
FESTGNLE	4.71	6.56	4.39				
FRSTGNLI	4.19	6.76	4.44				

**Table 5:** Comparison of performance between the additive method, SVRMHC, and SVR models with sparse encoding scheme for H2-Db, H2-Kb and H2-Kk.

		**Additive method**	**SVRMHC**	**SVR, Sparse encoding**
**H2-Db**	Numbers of outliers	6	3	5
	*q*^2^(LOO_CV)	0.401	0.456	0.459
**H2-Kb**	Numbers of outliers	7	6	8
	*q*^2^(LOO_CV)	0.454	0.486	0.352
**H2-Kk**	Numbers of outliers	2	0	1
	*q*^2^(LOO_CV)	0.456	0.721	0.523

**Table 6:** ROC-based comparison of the five predicting methods – SVRMHC, additive, SYFPEITHI, BIMAS, RANKPEP and SVMHC, after overlapped peptides were removed for SVRMHC and additive methods, but not for the four qualitative methods.

	**SVRMHC**	**Additive**	**BIMAS**	**SYFPEITHI**	**RANKPEP**	**SVMHC**
**H2-Db**	0.658	0.58	0.66	0.646	0.677	0.632
**H2-Kb**	0.83	0.766	0.769	0.731	0.485	0.748
